# Association between systemic inflammation indices and carotid plaques in acute coronary syndrome

**DOI:** 10.3389/fcvm.2026.1762926

**Published:** 2026-04-01

**Authors:** Mengmeng Ren, Yiming Wang, Lin Zhong, Chunxiao Wang

**Affiliations:** Department of Cardiology, The Affiliated Yantai Yuhuangding Hospital of Qingdao University, Yantai, Shandong, China

**Keywords:** acute coronary syndrome, carotid plaque, inflammation, MLR, PLR, SII

## Abstract

**Background:**

Ischemic stroke is a leading cause of death and disability worldwide. Identifying patients with carotid atherosclerosis who are at high risk is clinically important. Acute coronary syndrome (ACS) is a manifestation of systemic atherosclerosis, in which inflammation plays a key role in plaque progression and destabilization. However, the relationship between blood cell-derived systemic inflammation indices and carotid plaque burden in patients with ACS remains unclear.

**Methods:**

This retrospective study included 7,178 patients with ACS. Carotid ultrasound was used to classify patients into carotid plaque and non-plaque groups. Systemic inflammation indices—including neutrophil-to-lymphocyte ratio (NLR), monocyte-to-lymphocyte ratio (MLR), neutrophil-to-monocyte-plus-lymphocyte ratio (NMLR), systemic inflammatory response index (SIRI), systemic immune-inflammatory index (SII), aggregate index of systemic inflammation (AISI), and platelet-to-lymphocyte ratio (PLR)—were calculated from routine blood tests. Multivariable logistic regression, restricted cubic spline models, and subgroup analyses were used to evaluate their associations with carotid plaques.

**Results:**

Patients with carotid plaques had significantly higher NLR, MLR, NMLR, SIRI, SII, AISI, and PLR. After full adjustment, MLR, SIRI, and AISI showed significant non-linear associations with carotid plaques, with inflection points of 0.26, 0.9, and 205, respectively. Compared with the lowest quartile, the highest quartile was associated with increased odds of carotid plaques for MLR (OR 1.453), SIRI (OR 1.409), and AISI (OR 1.379). Subgroup analyses showed that these associations were mainly observed in women and in non-diabetic patients. In patients with hypertension, MLR, SIRI, and AISI remained positively associated with carotid plaques.

**Conclusion:**

MLR, SIRI, and AISI are independently associated with carotid plaques in patients with ACS, particularly among women and non-diabetic individuals, and may serve as potential markers for ischemic stroke risk stratification in this population.

## Introduction

Ischemic stroke is a common disease that seriously endangers human life and health and imposes a significant economic burden on society (1). Atherosclerosis is an important pathological basis of ischemic stroke, and vascular stenosis caused by internal carotid atherosclerosis accounts for 8%–15% of ischemic stroke cases (2). However, about 1%–2% of adults have no clinical symptoms of carotid artery stenosis, so early identification of a possible population with carotid artery stenosis is of great clinical importance. Acute coronary syndrome (ACS) shares a common pathological basis as stroke caused by atherosclerosis. Patients with ACS are usually associated with various diseases such as hypertension, diabetes, and dyslipidemia, all of which are risk factors leading to carotid plaque formation (3). ACS has recently been shown to increase the risk of perioperative ischemic stroke in non-cardiac surgery (4). Therefore, early screening of carotid plaque in patients with ACS is important for preventing stroke and improving the prognosis of ACS.

ACS represents two major clinical manifestations of systemic atherosclerotic disease. Carotid plaque, as an established marker of overall atherosclerotic burden, provides incremental prognostic information for future ischemic stroke and major adverse cardiovascular events, highlighting its important role in integrated cardiovascular and cerebrovascular risk assessment. Notably, polyvascular atherosclerosis, such as the concomitant involvement of coronary and carotid arteries, defines a particularly high-risk clinical phenotype with a substantially increased burden of ischemic events, underscoring the clinical importance of early carotid plaque screening in patients with ACS (5–8). Beyond lipid accumulation, atherosclerosis is driven by dysregulated inflammatory processes involving myeloid cell activation and cytokine signaling (9), as supported by randomized clinical trials of anti-inflammatory therapies, including IL-1β inhibition and colchicine, which have demonstrated a causal role of inflammation in recurrent ischemic events (10, 11). In this context, systemic inflammation indices derived from routine blood counts—such as the neutrophil-to-lymphocyte ratio (NLR), monocyte-to-lymphocyte ratio (MLR), systemic inflammatory response index (SIRI), systemic immune-inflammation index (SII), and aggregate index of systemic inflammation (AISI)—have been associated with carotid atherosclerosis and adverse clinical phenotypes in the general population and in patients with coronary artery disease (12–15). However, evidence specifically linking these blood cell-derived inflammatory indices to carotid plaque in patients with ACS remains limited and is largely based on small observational datasets, leaving uncertainty regarding which indices are most informative in this high-risk population and whether these associations vary across clinical subgroups (16). Moreover, in ACS, particularly among patients with ST-segment elevation myocardial infarction (STEMI), ischemia–reperfusion injury can markedly amplify systemic inflammatory signaling, including TNF-α–TNF receptor-1-related pathways, potentially influencing the progression of carotid atherosclerosis (17). Therefore, the present study aimed to comprehensively evaluate the associations between multiple blood count-derived systemic inflammation indices and carotid plaques in patients with ACS to support more refined cardiovascular and cerebrovascular risk stratification.

Numerous studies have demonstrated the critical role of inflammation in the development of atherosclerosis (18). In recent years, new indicators for evaluating systemic inflammation derived from the values of leukocytes, lymphocytes, monocytes, neutrophils, and platelets contained in blood routine—including NLR, MLR, NMLR, SIRI, SII, AISI, and PLR—have shown diagnostic and prognostic value in atherosclerosis-related diseases (14, 15). SII and NLR have been identified as strong predictors of stroke severity in large artery atherosclerosis (LAA) (12). Another study has found that new inflammatory indices SIRI, NLR, and LMR are associated with carotid atherosclerosis in middle-aged and elderly men (19). Several new indices of inflammation have also been demonstrated to correlate with severity, prognosis, and presence of ACS and acute myocardial infarction (20). However, studies on the association between blood cell count-derived systemic inflammation indices and carotid plaques in patients with ACS are lacking.

Notably, among patients with ACS, the subgroup with ST-segment elevation myocardial infarction (STEMI) exhibits pronounced ischemia–reperfusion injury and an enhanced systemic inflammatory response, which may uniquely influence the development of carotid atherosclerosis, as suggested by recent mechanistic studies linking TNF-α-mediated postreperfusion inflammation to vascular pathology (17).

Therefore, the present study aimed to analyze the correlation between systemic inflammation indices derived from blood cell counts and carotid plaques, which may provide a theoretical basis for the diagnosis, prevention, and treatment of carotid plaques in patients with ACS.

## Materials and methods

### Diagnostic criteria

ACS was diagnosed according to current clinical guidelines, based on typical ischemic chest pain, electrocardiographic changes (including ST-segment elevation, ST-segment depression, or T-wave inversion), and elevated biomarkers of myocardial injury, such as high-sensitivity cardiac troponin I. According to electrocardiographic findings and biochemical markers, ACS was further classified into ST-segment elevation myocardial infarction (STEMI) and non-ST-segment elevation ACS (NSTEMI or unstable angina). ACS was defined as either (1) acute myocardial infarction according to the Third Universal Definition of Myocardial Infarction, or (2) hospitalization for symptoms consistent with myocardial ischemia not fulfilling the above criteria but with evidence of coronary heart disease, including angiographic stenosis of ≥50% in at least one coronary artery, a history of coronary artery bypass grafting, or a history of percutaneous coronary intervention.

### Study population and design

This retrospective study included 7,178 patients with ACS who visited the Department of Cardiology, Yantai Yuhuangding Hospital between 2000 and 2025. All patients enrolled had confirmed ACS by coronary angiography and were older than 18 years of age. All patients had to undergo routine blood tests and carotid ultrasound. Exclusion criteria included severe liver or kidney dysfunction, previous stroke, malignant tumors, hematological diseases, autoimmune diseases, active infections, and other heart diseases (including acute decompensated heart failure, clinically significant arrhythmias, such as atrial fibrillation, and structural heart disease).

This study was approved by the Ethics Committee of Yuhuangding Hospital, Yantai (approval number 2024-203).

### Data collection

Clinical information was collected for all patients, including gender, age, smoking history, alcohol history, and histories of hypertension, diabetes, and hyperlipidemia. Fasting blood samples were collected within 24 h after admission. Assessments included complete blood routine (Sysmex XN-9000), biochemical tests (Stream SuperB-800), BNP, myocardial injury (hsTnI and CK-MB), and D-dimer tests. Collected indices included blood routine parameters (white blood cells, red blood cells, platelets, lymphocytes, monocytes, and neutrophils), biochemical values [AST, ALT, CREA, UA, CHOL, TG, LDL-C, HDL-C, Lp (a), GLU, and HCY], BNP, myocardial injury indices (hsTnI and CK-MB), and D-dimer.

### Calculation of systemic inflammation indices

According to peripheral blood cell counts, systemic inflammation indices were calculated as follows: NLR = neutrophils/lymphocytes; MLR = monocytes/lymphocytes; NMLR = (monocytes + neutrophils)/lymphocytes; SIRI = (neutrophils × monocytes)/lymphocytes; SII = neutrophils × platelets/lymphocytes; AISI = (neutrophils × platelets × monocytes)/lymphocytes; and PLR = platelets/lymphocytes.

### Statistical analyses

Model 1 was adjusted for baseline demographic characteristics and traditional cardiovascular risk factors, including gender, age, hypertension, diabetes, hyperlipidemia, smoking status, and alcohol consumption. Model 2 was further adjusted for biochemical parameters reflecting metabolic status and organ function, including aspartate aminotransferase (AST), alanine aminotransferase (ALT), creatinine (CREA), uric acid (UA), total cholesterol (TC), triglycerides (TG), low-density lipoprotein cholesterol (LDL-C), high-density lipoprotein cholesterol (HDL-C), lipoprotein(a) [Lp(a)], and glucose (GLU). Model 3 was additionally adjusted for biomarkers reflecting the severity of acute coronary syndrome, including B-type natriuretic peptide (BNP), high-sensitivity cardiac troponin I (hs-TnI), creatine kinase-MB (CK-MB), and D-dimer.

Kolmogorov–Smirnov tests and Q–Q plots were applied to test the normality of quantitative variables. Variables that met normality were described by means ± standard deviations and analyzed for differences according to the *t*-test. Variables that did not meet normality were described by medians and quartiles and analyzed for differences according to the Mann–Whitney *U* test. Qualitative variables were subjected to chi-square tests and described according to frequency and constituent ratio. Violin plots were drawn to describe the levels of inflammatory indices in both groups of patients. Restrictive cubic spline plots were applied to analyze the non-linear relationship between systemic inflammatory indices and carotid plaques. Univariate and multivariate logistic regression models were constructed to assess the association between systemic inflammatory indices and carotid plaques, and the size of the association effect was assessed based on ORs and their 95% confidence intervals. Statistical analyses were conducted using software R 4.4.1 and IBM SPSS 26. The test level was set at *α* = 0.05. Missing data were handled as follows: Variables with less than 30% missingness were imputed using a random forest-based imputation method with a maximum of 50 iterations. Variables with missingness exceeding 30% were excluded from subsequent analyses. All imputations were performed prior to model fitting.

## Results

### Baseline characteristics

A total of 7,178 patients with ACS were included in this study, and the proportion of women was 44.1%, with a mean age of 65.74 ± 10.10 years. Of these, 6,031 patients had carotid plaques and 1,147 did not. Patients with carotid plaques were older, more frequently men, had higher prevalences of hypertension, diabetes, and hyperlipidemia, and had a greater number of people who smoked and consumed alcohol. Meanwhile, there were significant differences in ALT, CREA, UA, TC, TG, HDL-C, LP (a), GLU, HCY, BNP, hsTnI, CK-MB, D-dimer, WBC, RBC, LYM, MONO, NEUT, and PLT between the two groups (Table 1).

**Table 1:** Baseline characteristics of patients with acute coronary syndrome stratified by carotid plaque status.

Variables	Total (*n* = 7,178)	Without carotid plaque (*n* = 1,147)	With carotid plaque (*n* = 6,031)	*P*
Gender (*n*, %)				<0.001*
Female	3,167 (44.1)	683 (59.5)	2,484 (41.2)	
Male	4,011 (55.9)	464 (40.5)	3,547 (58.8)	
Age (years)	65.74 ± 10.10	60.00 ± 9.98	66.83 ± 9.74	<0.001*
Hypertension (*n*, %)	5,370 (74.8)	743 (64.8)	4,627 (76.7)	<0.001*
Diabetes (*n*, %)	2,739 (38.2)	365 (31.8)	2,374 (39.4)	<0.001*
Hyperlipidemia (*n*, %)	1,097 (15.3)	201 (17.5)	896 (14.9)	0.021*
Smoke (*n*, %)	2,249 (31.3)	237 (20.7)	2,012 (33.4)	<0.001*
Drink (*n*, %)	1,870 (26.1)	207 (18.0)	1,663 (27.6)	<0.001*
WBC (×10^12^/L)	6.13 (5.12, 7.43)	5.90 (4.92, 7.05)	6.18 (5.16, 7.49)	<0.001*
RBC (×10^9^/L)	4.49 ± 0.58	4.56 ± 0.55	4.47 ± 0.58	<0.001*
LYM (×10^9^/L)	1.84 ± 0.66	1.91 ± 0.63	1.82 ± 0.67	<0.001*
MONO (×10^9^/L)	0.47 (0.38, 0.59)	0.44 (0.35, 0.55)	0.48 (0.39, 0.60)	<0.001*
NEUT (×10^9^/L)	3.57 (2.78, 4.61)	3.31 (2.57, 4.31)	3.61 (2.82, 4.67)	<0.001*
PLT (×10^9^/L)	216.00 (181.00, 256.00)	219.00 (187.00, 260.00)	216.00 (180.00, 256.00)	0.010*
AST (U/L)	21.00 (17.00, 26.00)	21.00 (17.00, 26.00)	21.000 (17.00, 26.00)	0.621
ALT (U/L)	18.00 (13.00, 26.00)	18.00 (14.00, 27.00)	18 (13.00, 26.00)	0.004*
CREA (µmol/L)	63.00 (53.00, 76.00)	59.00 (51.00, 70.00)	64.00 (54.00, 77.00)	<0.001*
UA (µmol/L)	342.48 ± 100.08	329.92 ± 93.04	344.87 ± 101.19	<0.001*
CHOL (mmol/L)	4.74 ± 1.33	4.82 ± 1.28	4.73 ± 1.34	0.044*
TG (mmol/L)	1.23 (0.92, 1.73)	1.25 (0.94, 1.85)	1.23 (0.91, 1.71)	0.045*
LDL-C (mmol/L)	2.87 ± 1.05	2.86 ± 1.01	2.87 ± 1.06	0.705
HDL-C (mmol/L)	1.22 ± 0.32	1.25 ± 0.32	1.21 ± 0.32	<0.001*
LP (a) (mg/L)	171.00 (81.00, 336.00)	140.00 (72.00, 282.00)	177.00 (84.00, 348.00)	<0.001*
GLU (mmol/L)	5.50 (4.88, 6.82)	5.36 (4.79, 6.37)	5.53 (4.90, 6.89)	<0.001*
HCY (µmol/L)	12.60 (10.70, 15.30)	11.50 (10.00, 13.80)	12.80 (10.90, 15.60)	<0.001*
BNP (pg/mL)	53.00 (23.82, 135.00)	36.73 (18.12, 90.29)	56.77 (25.32, 143.84)	<0.001*
hsTnI (pg/mL)	5.94 (2.40, 25.90)	3.50 (1.50, 12.65)	6.50 (2.60, 27.96)	<0.001*
CK-MB (pg/mL)	1.24 (0.80, 2.08)	1.10 (0.70, 1.80)	1.30 (0.80, 2.10)	<0.001*
D-dimer (mg/L)	0.57 (0.19, 0.89)	0.50 (0.18, 0.77)	0.58 (0.19, 0.90)	<0.001*

WBC, white blood cell; RBC, red blood cell; LYM, lymphocyte; MONO, monocyte; NEUT, neutrophils; PLT, platelets; AST, aspartate aminotransferase; ALT, alanine aminotransferase; CREA, creatinine; UA, uric acid; CHOL, cholesterol; TG, triglyceride; LDL-C, low-density lipoprotein cholesterol; HDL-C, high-density lipoprotein cholesterol; LP (a), lipoprotein a; GLU, blood glucose; HCY, homocysteines; BNP, B-type natriuretic peptide; hsTnI, high-sensitivity troponin I; CK-MB, creatine Kinase-MB.

* Indicates statistical significance.

### Correlation of systemic inflammation indices with Baseline characteristics

 Systemic inflammation indices were calculated and compared between the two groups. It was found that all inflammation-related indices—NLR, MLR, NMLR, SIRI, SII, AISI, and PLR—were higher in patients with carotid plaques (Table 2). Correlation analyses between systemic inflammation indices and baseline characteristics are presented in the Supplementary Table. Overall, most indices exhibited weak to moderate correlations with demographic factors, cardiometabolic risk factors, and laboratory parameters. In general, NLR, MLR, NMLR, SIRI, SII, and AISI were positively correlated with age, female sex, renal function markers, and myocardial injury biomarkers, whereas inverse correlations were observed with lipid-related variables, including total cholesterol, triglycerides, LDL-C, and HDL-C. PLR showed a distinct correlation pattern, being more strongly related to age and myocardial injury markers, and inversely associated with metabolic and inflammatory conditions. Importantly, none of the correlations reached levels indicative of substantial collinearity, supporting the stability of subsequent multivariable models.

**Table 2:** Systemic inflammation parameters in different group.

Variables	Total (*n* = 7,178)	With carotid plaques (*n* = 1,147)	Without carotid plaques (*n* = 6,031)	*P*
NLR	1.96 (1.44, 2.84)	1.75 (1.32, 2.47)	2.01 (1.47, 2.92)	<0.001*
MLR	0.26 (0.20, 0.35)	0.24 (0.18, 0.31)	0.27 (0.21, 0.36)	<0.001*
NMLR	2.24 (1.66, 3.18)	2.00 (1.52, 2.76)	2.29 (1.70, 3.29)	<0.001*
SIRI	0.92 (0.61, 1.48)	0.77 (0.51, 1.18)	0.96 (0.63, 1.55)	<0.001*
SII	424.86 (296.13, 640.91)	390.70 (273.38, 568.74)	434.30 (300.27, 655.51)	<0.001*
AISI	200.10 (125.02, 336.03)	172.30 (106.22, 276.25)	206.54 (130.26, 349.64)	<0.001*
PLR	122.01 (95.21, 159.35)	118.30 (92.77, 153.42)	122.73 (95.61, 160.52)	0.006*

NLR, neutrophil-to-lymphocyte ratio; MLR, monocyte-to-lymphocyte ratio; NMLR, neutrophil-to-monocyte-to-lymphocyte ratio; SIRI, systemic inflammation response index; SII, systemic immune-inflammation index; AISI, aggregate index of systemic inflammation; PLR, platelet-to-lymphocyte ratio.

* Indicates statistical significance.

### Correlation of systemic inflammation indices with carotid plaque

**Figure 1:**
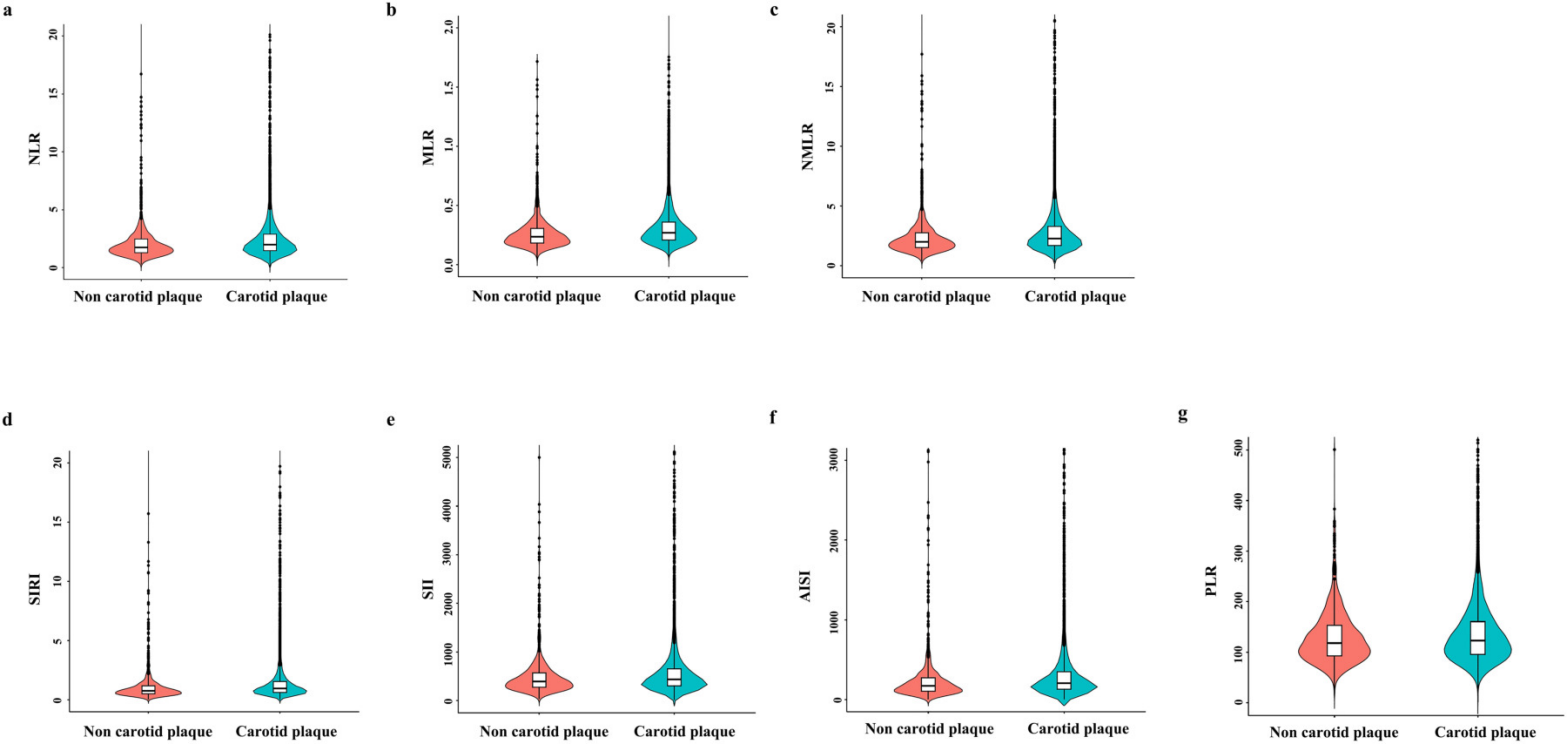
Distribution of systemic inflammation indices in patients with ACS with and without carotid plaques. **(a)** Neutrophil-to-lymphocyte ratio (NLR); **(b)** monocyte-to-lymphocyte ratio (MLR); **(c)** neutrophil-monocyte-to-lymphocyte ratio (NMLR); **(d)** systemic inflammatory response index (SIRI); **(e)** systemic immune-inflammation index (SII); **(f)** aggregate index of systemic inflammation (AISI); and **(g)** platelet-to-lymphocyte ratio (PLR). Red and blue violin plots represent patients without and with carotid plaques, respectively.

**Figure 2:**
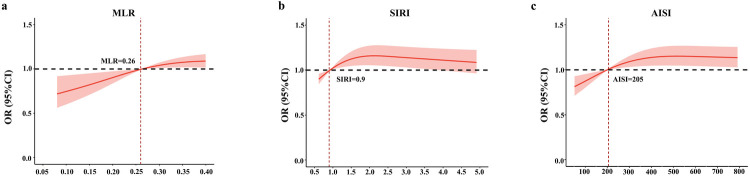
Restricted cubic spline curves for the associations between systemic inflammation indices and carotid plaques in patients with ACS. **(a)** Monocyte-to-lymphocyte ratio (MLR); **(b)** systemic inflammatory response index (SIRI); and **(c)** aggregate index of systemic inflammation (AISI). The solid line indicates the adjusted odds ratio (OR) for carotid plaques with 95% confidence interval (shaded area); the horizontal dashed line represents OR = 1.0; the vertical dashed line indicates the inflection point (cut-off) of each index.

Figure 1 shows the distribution of systemic inflammation indices in patients with and without carotid plaques. Next, the association between systemic inflammation indices and carotid plaques was assessed. As shown in Table 3, systemic inflammation indices were converted from continuous variables to categorical variables (Q1–Q4), and NLR, MLR, NMLR, SIRI, SII, AISI, PLR were positively correlated with carotid plaques without adjusting for covariates (*p* < 0.05). In Model 1, all indices except PLR were positively associated with carotid plaques (*p* < 0.05). In Model 2 and Model 3, only MLR, SIRI, and AISI were positively associated with carotid plaques (*p* < 0.05). Sensitivity analysis in Model 3 showed that the highest MLR, SIRI, and AISI quartiles (Q4 vs. Q1) (Q4) had 1.453, 1.409, and 1.379-fold increased odds of developing carotid plaques, respectively, compared with the lowest quartiles (Q4 vs. Q1) (Q1) (*p* < 0.05). Figure 2 shows a non-linear association between MLR, SIRI, and AISI and carotid plaques with inflection points of 0.26, 0.9, and 205, respectively, after adjusting for all confounders. The identified inflection points (0.26 for MLR, 0.9 for SIRI, and 205 for AISI) were interpreted as threshold values at which the direction of association changed. Specifically, when MLR was below 0.26, increasing MLR was associated with a decreased risk of carotid plaques (OR < 1), whereas when MLR exceeded 0.26, higher MLR was associated with an increased risk of carotid plaques (OR > 1).

**Table 3:** Association between systemic inflammation parameters and baseline characteristics.

Variables	NLR		MLR		NMLR		SIRI		SII		AISI		PLR	*P*
	*r*	*P*	*r*	*P*	*r*	*P*	*r*	*P*	*r*	*P*	*r*	*P*	*r*	
Gender, F/M	0.204	<0.001***	0.305	<0.001***	0.218	<0.001***	0.283	<0.001***	0.091	<0.001***	0.185	<0.001***	−0.032	0.007**
Age	0.132	<0.001***	0.171	<0.001***	0.139	<0.001***	0.115	<0.001***	0.076	<0.001***	0.073	<0.001***	0.102	<0.001***
Hypertension	0.038	0.001**	0.004	0.745	0.035	0.003**	0.044	<0.001***	0.062	<0.001***	0.061	<0.001***	0.022	0.06
Diabetes	−0.017	0.158	−0.052	<0.001***	−0.021	0.077	0.007	0.539	−0.001	0.926	0.016	0.184	−0.063	<0.001***
Hyperlipidemia	−0.089	<0.001***	−0.093	<0.001***	−0.091	<0.001***	−0.075	<0.001***	−0.063	<0.001***	−0.058	<0.001***	−0.067	<0.001***
Smoking	0.108	<0.001***	0.168	<0.001***	0.117	<0.001***	0.189	<0.001***	0.059	<0.001***	0.142	<0.001***	−0.06	<0.001***
Drinking	0.073	<0.001***	0.142	<0.001***	0.081	<0.001***	0.136	<0.001***	0.03	0.010*	0.096	<0.001***	−0.04	0.001**
AST	0.013	0.281	0.067	<0.001***	0.019	0.111	0.044	<0.001***	−0.022	0.059	0.015	0.218	−0.042	<0.001***
ALT	−0.02	0.093	−0.005	0.696	−0.019	0.106	0.029	0.014*	−0.029	0.015*	0.017	0.146	−0.1	<0.001***
CREA	0.199	<0.001***	0.253	<0.001***	0.208	<0.001***	0.257	<0.001***	0.126	<0.001***	0.192	<0.001***	0.014	0.248
UA	0.026	0.031*	0.064	<0.001***	0.029	0.012*	0.101	<0.001***	−0.006	0.628	0.068	<0.001***	−0.113	<0.001***
TC	−0.142	<0.001***	−0.198	<0.001***	−0.151	<0.001***	−0.151	<0.001***	−0.037	0.002**	−0.07	<0.001***	−0.005	0.699
TG	−0.141	<0.001***	−0.184	<0.001***	−0.149	<0.001***	−0.092	<0.001***	−0.063	<0.001***	−0.04	0.001**	−0.134	<0.001***
LDL-C	−0.096	<0.001***	−0.14	<0.001***	−0.103	<0.001***	−0.099	<0.001***	−0.005	0.691	−0.029	0.015*	0.018	0.137
HDL-C	−0.11	<0.001***	−0.153	<0.001***	−0.116	<0.001***	−0.16	<0.001***	−0.063	<0.001***	−0.116	<0.001***	0.019	0.104
LP(a)	0.065	<0.001***	0.045	<0.001***	0.064	<0.001***	0.056	<0.001***	0.102	<0.001***	0.083	<0.001***	0.101	<0.001***
GLU	0.086	<0.001***	−0.014	0.245	0.078	<0.001***	0.075	<0.001***	0.094	<0.001***	0.083	<0.001***	<0.001	0.973
HCY	0.191	<0.001***	0.227	<0.001***	0.199	<0.001***	0.23	<0.001***	0.137	<0.001***	0.181	<0.001***	0.055	<0.001***
BNP	0.177	<0.001***	0.222	<0.001***	0.185	<0.001***	0.196	<0.001***	0.111	<0.001***	0.142	<0.001***	0.068	<0.001***
hsTnI	0.226	<0.001***	0.247	<0.001***	0.233	<0.001***	0.263	<0.001***	0.162	<0.001***	0.209	<0.001***	0.05	<0.001***
CK-MB	0.126	<0.001***	0.139	<0.001***	0.13	<0.001***	0.145	<0.001***	0.077	<0.001***	0.105	<0.001***	0.018	0.128
D-dimer	0.186	<0.001***	0.175	<0.001***	0.189	<0.001***	0.178	<0.001***	0.163	<0.001***	0.16	<0.001***	0.125	<0.001***

WBC, white blood cell; RBC, red blood cell; LYM, lymphocyte; MONO, monocyte; NEUT, neutrophils; PLT, platelets; AST, Aspartate aminotransferase; ALT, Alanine Aminotransferase; CREA, Creatinine; UA, uric acid; CHOL, cholesterol; TG, triglyceride; LDL-C, low-density lipoprotein cholesterol; HDL-C, high-density lipoprotein cholesterol; LP (a), lipoprotein a; GLU, blood glucose; HCY, homocysteines; BNP, B-type natriuretic peptide; hsTnI, high-sensitivity Troponin I; CK-MB, Creatine Kinase-MB.

***: *P* < 0.001; **: *P* < 0.01; *: *P* < 0.05.

### Subgroup analyses

After adjustment for all confounders, subgroup analyses were performed by gender, hypertension, and diabetes. Although MLR, SIRI, and AISI were positively associated with carotid plaques, they were not consistently associated with carotid plaques in different subgroups. In the gender subgroup analysis, MLR, SIRI, and AISI were positively associated with carotid plaques only in female patients (*p* < 0.05) and not in male patients (Table 4). In the diabetes subgroup analysis, MLR, SIRI, and AISI were positively associated with carotid plaque only in non-diabetic patients (*p* < 0.05) and not in diabetic patients (Table 5). In the hypertension subgroup analysis, MLR was positively associated with carotid plaques in both hypertensive and non-hypertensive patients, whereas SIRI and AISI were positively associated with carotid plaques only in hypertensive patients (*p* < 0.05) and not in non-hypertensive patients (Table 6). Subgroup analyses showed that these associations were mainly observed in women and in nondiabetic patients. In patients with hypertension, MLR, SIRI, and AISI remained positively associated with carotid plaques (Table 7).

**Table 4:** Association between systemic inflammation parameters and carotid plaque.

Inflammatory parameter	Quartile 1		Quartile 2		Quartile 3		Quartile 4		*P*_trend_
	OR (95% CI)	*P*	OR (95% CI)	*P*	OR (95% CI)	*P*	OR (95% CI)	*P*	
NLR									
Range	≤1.44		1.44 < NLR ≤ 1.96		1.96 < NLR ≤ 2.84		>2.84		
Unadjusted	1 [Reference]	—	1.184 (1.003, 1.399)	0.046	1.561 (1.310, 1.860)	<0.001	2.052 (1.703, 2.472)	<0.001	<0.001
Model 1	1 [Reference]	—	1.037 (0.867, 1.239)	0.693	1.191 (0.986, 1.440)	0.070	1.305 (1.067, 1.595)	0.010	0.034
Model 2	1 [Reference]	—	1.012 (0.845, 1.212)	0.897	1.150 (0.949, 1.394)	0.155	1.240 (1.005, 1.530)	0.045	0.134
Model 3	1 [Reference]	—	1.012 (0.845, 1.212)	0.900	1.145 (0.944, 1.388)	0.169	1.219 (0.987, 1.506)	0.067	0.187
MLR									
Range	≤0.20		0.20 < MLR ≤ 0.26		0.26 < MLR ≤ 0.35		>0.35		
Unadjusted	1 [Reference]	—	1.402 (1.187, 1.655)	<0.001	1.767 (1.487, 2.099)	<0.001	2.702 (2.231, 3.271)	<0.001	<0.001
Model 1	1 [Reference]	—	1.189 (0.994, 1.420)	0.058	1.166 (0.964, 1.410)	0.113	1.509 (1.223, 1.862)	<0.001	0.002
Model 2	1 [Reference]	—	1.166 (0.974, 1.396)	0.095	1.142 (0.942, 1.385)	0.177	1.483 (1.192, 1.846)	<0.001	0.006
Model 3	1 [Reference]	—	1.165 (0.973, 1.394)	0.098	1.137 (0.937, 1.379)	0.193	1.453 (1.165, 1.811)	0.001	0.011
NMLR									
Range	≤1.66		1.66 < NMLR ≤ 2.24		2.24 < NMLR ≤ 3.18		>3.18		
Unadjusted	1 [Reference]	—	1.215 (1.029, 1.433)	0.021	1.595 (1.339, 1.900)	<0.001	2.161 (1.791, 2.606)	<0.001	<0.001
Model 1	1 [Reference]	—	1.073 (0.898, 1.282)	0.437	1.193 (0.986, 1.442)	0.069	1.348 (1.101, 1.650)	0.004	0.024
Model 2	1 [Reference]	—	1.043 (0.871, 1.248)	0.646	1.151 (0.949, 1.396)	0.152	1.287 (1.042, 1.590)	0.019	0.097
Model 3	1 [Reference]	—	1.043 (0.871, 1.248)	0.649	1.144 (0.943, 1.388)	0.171	1.264 (1.022, 1.565)	0.031	0.144
SIRI									
Range	≤0.61		0.61 < SIRI≤0.92		0.92 < SIRI≤1.48		>1.48		
Unadjusted	1 [Reference]	—	1.450 (1.229, 1.712)	<0.001	1.799 (1.514, 2.138)	<0.001	2.601 (2.155, 3.141)	<0.001	<0.001
Model 1	1 [Reference]	—	1.197 (1.001, 1.431)	0.048	1.266 (1.048, 1.531)	0.015	1.526 (1.241, 1.875)	<0.001	0.001
Model 2	1 [Reference]	—	1.156 (0.966, 1.385)	0.114	1.209 (0.997, 1.465)	0.053	1.435 (1.157, 1.779)	0.001	0.011
Model 3	1 [Reference]	—	1.158 (0.967, 1.387)	0.110	1.202 (0.991, 1.457)	0.061	1.409 (1.134, 1.750)	0.002	0.019
SII									
Range	≤296.13		296.13 < SII≤424.86		424.86 < SII≤640.91		>640.91		
Unadjusted	1 [Reference]	—	1.137 (0.959, 1.348)	0.139	1.296 (1.089, 1.543)	0.003	1.676 (1.395, 2.014)	<0.001	<0.001
Model 1	1 [Reference]	—	1.109 (0.926, 1.330)	0.265	1.217 (1.010, 1.466)	0.039	1.299 (1.067, 1.581)	0.009	0.046
Model 2	1 [Reference]	—	1.073 (0.893, 1.289)	0.452	1.162 (0.962, 1.403)	0.120	1.190 (0.972, 1.457)	0.093	0.290
Model 3	1 [Reference]	—	1.076 (0.896, 1.293)	0.431	1.162 (0.962, 1.404)	0.120	1.177 (0.960, 1.443)	0.117	0.329
AISI									
Range	≤125.02		125.02 < AISI≤200.10		200.10 < AISI≤336.03		>336.03		
Unadjusted	1 [Reference]	—	1.463 (1.235, 1.734)	<0.001	1.513 (1.276, 1.794)	<0.001	2.194 (1.823, 2.640)	<0.001	<0.001
Model 1	1 [Reference]	—	1.349 (1.126, 1.618)	0.001	1.295 (1.077, 1.558)	0.006	1.523 (1.247, 1.860)	<0.001	<0.001
Model 2	1 [Reference]	—	1.320 (1.100, 1.585)	0.003	1.234 (1.024, 1.487)	0.027	1.399 (1.139, 1.719)	0.001	0.003
Model 3	1 [Reference]	—	1.323 (1.102, 1.588)	0.003	1.235 (1.024, 1.488)	0.027	1.379 (1.121, 1.697)	0.002	0.004
PLR									
Range	≤95.21		95.21 < PLR ≤ 122.01		122.01 < PLR ≤ 159.35		>159.35		
Unadjusted	1 [Reference]	—	1.087 (0.912, 1.295)	0.352	1.073 (0.901, 1.278)	0.427	1.300 (1.085, 1.558)	0.004	0.036
Model 1	1 [Reference]	—	1.124 (0.932, 1.354)	0.222	1.101 (0.912, 1.327)	0.316	1.227 (1.010, 1.492)	0.039	0.228
Model 2	1 [Reference]	—	1.114 (0.923, 1.345)	0.260	1.075 (0.889, 1.299)	0.454	1.174 (0.962, 1.435)	0.115	0.439
Model 3	1 [Reference]	—	1.118 (0.925, 1.350)	0.248	1.075 (0.889, 1.299)	0.457	1.163 (0.952, 1.422)	0.139	0.477

NLR, neutrophil-to-lymphocyte ratio; MLR, monocyte-to-lymphocyte ratio; NMLR, neutrophil-to-monocyte-to-lymphocyte ratio; SIRI, systemic inflammation response index; SII, systemic immune-inflammation index; AISI, aggregate index of systemic inflammation; PLR, platelet-to-lymphocyte ratio.

**Table 5:** Gender subgroup analysis of the association between systemic inflammation parameters and carotid plaque.

Inflammatory parameter	Quartile 1		Quartile 2		Quartile 3		Quartile 4	
	OR (95% CI)	*P*	OR (95% CI)	*P*	OR (95% CI)	*P*	OR (95% CI)	*P*
NLR								
Range	≤1.44		1.44 < NLR ≤ 1.96		1.96 < NLR ≤ 2.84		>2.84	
Female	1 [Reference]	—	1.007 (0.801, 1.266)	0.950	1.179 (0.910, 1.526)	0.213	1.321 (0.981, 1.778)	0.066
Male	1 [Reference]	—	0.975 (0.720, 1.320)	0.869	1.040 (0.767, 1.411)	0.801	1.039 (0.753, 1.434)	0.816
MLR								
Range	≤0.20		0.20 < MLR ≤ 0.26		0.26 < MLR ≤ 0.35		>0.35	
Female	1 [Reference]	—	1.126 (0.901, 1.407)	0.297	1.103 (0.850, 1.431)	0.461	1.558 (1.122, 2.163)	0.008
Male	1 [Reference]	—	1.170 (0.852, 1.606)	0.333	1.093 (0.802, 1.490)	0.573	1.284 (0.923, 1.785)	0.138
NMLR								
Range	≤1.66		1.66 < NMLR ≤ 2.24		2.24 < NMLR ≤ 3.18		>3.18	
Female	1 [Reference]	—	1.025 (0.816, 1.287)	0.832	1.110 (0.856, 1.439)	0.431	1.392 (1.028, 1.884)	0.032
Male	1 [Reference]	—	1.014 (0.750, 1.373)	0.926	1.106 (0.816, 1.500)	0.516	1.085 (0.787, 1.497)	0.617
SIRI								
Range	≤0.61		0.61 < SIRI≤0.92		0.92 < SIRI≤1.48		>1.48	
Female	1 [Reference]	—	1.033 (0.826, 1.293)	0.774	1.222 (0.937, 1.595)	0.138	1.492 (1.080, 2.061)	0.015
Male	1 [Reference]	—	1.341 (0.980, 1.837)	0.067	1.142 (0.845, 1.543)	0.387	1.328 (0.965, 1.829)	0.082
SII								
Range	≤296.13		296.13 < SII≤424.86		424.86 < SII≤640.91		>640.91	
Female	1 [Reference]	—	1.141 (0.897, 1.451)	0.284	1.210 (0.939, 1.559)	0.141	1.177 (0.891, 1.555)	0.251
Male	1 [Reference]	—	0.988 (0.740, 1.320)	0.937	1.039 (0.777, 1.389)	0.795	1.076 (0.791, 1.465)	0.641
AISI								
Range	≤125.02		125.02 < AISI≤200.10		200.10 < AISI≤336.03		>336.03	
Female	1 [Reference]	—	1.468 (1.158, 1.860)	0.001	1.179 (0.922, 1.508)	0.190	1.449 (1.075, 1.954)	0.015
Male	1 [Reference]	—	1.091 (0.810, 1.471)	0.566	1.180 (0.875, 1.592)	0.278	1.172 (0.862, 1.593)	0.312
PLR								
Range	≤95.21		95.21 < PLR ≤ 122.01		122.01 < PLR ≤ 159.35		>159.35	
Female	1 [Reference]	—	1.181 (0.912, 1.531)	0.208	1.113 (0.860, 1.439)	0.417	1.195 (0.912, 1.567)	0.196
Male	1 [Reference]	—	1.022 (0.774, 1.350)	0.876	1.028 (0.773, 1.369)	0.847	1.030(0.759, 1.398)	0.848

NLR, neutrophil-to-lymphocyte ratio; MLR, monocyte-to-lymphocyte ratio; NMLR, neutrophil-to-monocyte-to-lymphocyte ratio; SIRI, systemic inflammation response index; SII, systemic immune-inflammation index; AISI, aggregate index of systemic inflammation; PLR, platelet-to-lymphocyte ratio.

**Table 6:** Diabetes subgroup analysis of the association between systemic inflammation parameters and carotid plaque.

Inflammatory parameter	Quartile 1		Quartile 2		Quartile 3		Quartile 4	
	OR (95% CI)	*P*	OR (95% CI)	*P*	OR (95% CI)	*P*	OR (95% CI)	*P*
NLR								
Range	≤1.44		1.44 < NLR ≤ 1.96		1.96 < NLR ≤ 2.84		>2.84	
Non-diabetes	1 [Reference]	—	1.041 (0.831, 1.304)	0.729	1.218 (0.955, 1.554)	0.112	1.190 (0.915, 1.548)	0.194
Diabetes	1 [Reference]	—	0.946 (0.696, 1.285)	0.721	1.007 (0.730, 1.389)	0.968	1.213 (0.838, 1.756)	0.305
MLR								
Range	≤0.20		0.20 < MLR ≤ 0.26		0.26 < MLR ≤ 0.35		>0.35	
Non-diabetes	1 [Reference]	—	1.151 (0.916, 1.445)	0.228	1.098 (0.863, 1.397)	0.446	1.469 (1.117, 1.932)	0.006
Diabetes	1 [Reference]	—	1.179 (0.874, 1.591)	0.281	1.194 (0.858, 1.662)	0.294	1.397 (0.955, 2.044)	0.085
NMLR								
Range	≤1.66		1.66 < NMLR ≤ 2.24		2.24 < NMLR ≤ 3.18		>3.18	
Non-diabetes	1 [Reference]	—	1.097 (0.875, 1.374)	0.422	1.204 (0.944, 1.536)	0.135	1.226 (0.942, 1.594)	0.130
Diabetes	1 [Reference]	—	0.927 (0.685, 1.255)	0.624	1.025 (0.742, 1.416)	0.882	1.280 (0.877, 1.868)	0.200
SIRI								
Range	≤0.61		0.61 < SIRI≤0.92		0.92 < SIRI≤1.48		>1.48	
Non-diabetes	1 [Reference]	—	1.363 (1.085, 1.713)	0.008	1.243 (0.978, 1.579)	0.075	1.427 (1.090, 1.869)	0.010
Diabetes	1 [Reference]	—	0.863 (0.639, 1.165)	0.336	1.069 (0.765, 1.493)	0.697	1.302 (0.892, 1.902)	0.172
SII								
Range	≤296.13		296.13 < SII≤424.86		424.86 < SII≤640.91		>640.91	
Non-diabetes	1 [Reference]	—	1.163 (0.925, 1.462)	0.195	1.210 (0.957, 1.531)	0.112	1.206 (0.936, 1.556)	0.148
Diabetes	1 [Reference]	—	0.914 (0.668, 1.251)	0.575	1.047 (0.756, 1.450)	0.783	1.021 (0.718, 1.452)	0.906
AISI								
Range	≤125.02		125.02 < AISI≤200.10		200.10 < AISI≤336.03		>336.03	
Non-diabetes	1 [Reference]	—	1.444 (1.150, 1.813)	0.002	1.414 (1.119, 1.788)	0.004	1.416 (1.093, 1.834)	0.008
Diabetes	1 [Reference]	—	1.103 (0.805, 1.511)	0.542	0.923 (0.672, 1.269)	0.623	1.215 (0.846, 1.743)	0.291
PLR								
Range	≤95.21		95.21 < PLR ≤ 122.01		122.01 < PLR ≤ 159.35		>159.35	
Non-diabetes	1 [Reference]	—	1.090 (0.856, 1.388)	0.486	0.970 (0.766, 1.229)	0.804	1.158 (0.900, 1.490)	0.254
Diabetes	1 [Reference]	—	1.134 (0.836, 1.538)	0.419	1.256 (0.902, 1.749)	0.178	1.097(0.782, 1.540)	0.592

OR, odds ratio; CI, confidence interval; NLR, neutrophil-to-lymphocyte ratio; MLR, monocyte-to-lymphocyte ratio; NMLR, neutrophil-to-monocyte-to-lymphocyte ratio; SIRI, systemic inflammation response index; SII, systemic immune-inflammation index; AISI, aggregate index of systemic inflammation; PLR, platelet-to-lymphocyte ratio.

**Table 7:** Hypertension subgroup analysis of the association between systemic inflammation parameters and carotid plaque.

Inflammatory parameter	Quartile 1		Quartile 2		Quartile 3		Quartile 4	
	OR (95% CI)	*P*	OR (95% CI)	*P*	OR (95% CI)	*P*	OR (95% CI)	*P*
NLR								
Range	≤1.44		1.44 < NLR ≤ 1.96		1.96 < NLR ≤ 2.84		>2.84	
Non-HTN	1 [Reference]	—	1.100 (0.802, 1.510)	0.553	1.171 (0.822, 1.669)	0.381	1.167 (0.790, 1.723)	0.439
HTN	1 [Reference]	—	0.963 (0.772, 1.202)	0.739	1.120 (0.888, 1.412)	0.339	1.217 (0.944, 1.568)	0.129
MLR								
Range	≤0.20		0.20 < MLR ≤ 0.26		0.26 < MLR ≤ 0.35		>0.35	
Non-HTN	1 [Reference]	—	1.436 (1.032, 1.998)	0.032	1.055 (0.744, 1.496)	0.765	1.492 (1.005, 2.217)	0.047
HTN	1 [Reference]	—	1.052 (0.848, 1.305)	0.647	1.172 (0.927, 1.481)	0.185	1.384 (1.061, 1.805)	0.017
NMLR								
Range	≤1.66		1.66 < NMLR ≤ 2.24		2.24 < NMLR ≤ 3.18		>3.18	
Non-HTN	1 [Reference]	—	1.153 (0.841, 1.582)	0.377	1.173 (0.824, 1.669)	0.377	1.263 (0.853, 1.870)	0.244
HTN	1 [Reference]	—	0.986 (0.791, 1.229)	0.899	1.120 (0.888, 1.414)	0.339	1.235 (0.957, 1.594)	0.105
SIRI								
Range	≤0.61		0.61 < SIRI≤0.92		0.92 < SIRI≤1.48		>1.48	
Non-HTN	1 [Reference]	—	1.195 (0.867, 1.646)	0.277	1.117 (0.788, 1.583)	0.534	1.496 (1.001, 2.237)	0.050
HTN	1 [Reference]	—	1.132 (0.909, 1.409)	0.269	1.222 (0.968, 1.541)	0.092	1.340 (1.035, 1.736)	0.026
SII								
Range	≤296.13		296.13 < SII≤424.86		424.86 < SII≤640.91		>640.91	
Non-HTN	1 [Reference]	—	1.099 (0.802, 1.505)	0.558	1.310 (0.927, 1.852)	0.126	1.164 (0.799, 1.696)	0.429
HTN	1 [Reference]	—	1.065 (0.848, 1.338)	0.587	1.097 (0.873, 1.378)	0.428	1.152 (0.903, 1.471)	0.255
AISI								
Range	≤125.02		125.02 < AISI≤200.10		200.10 < AISI≤336.03		>336.03	
Non-HTN	1 [Reference]	—	1.488 (1.081, 2.049)	0.015	1.307 (0.931, 1.835)	0.122	1.388 (0.945, 2.040)	0.095
HTN	1 [Reference]	—	1.248 (0.997, 1.563)	0.053	1.182 (0.943, 1.481)	0.147	1.329 (1.037, 1.702)	0.024
PLR								
Range	≤95.21		95.21 < PLR ≤ 122.01		122.01 < PLR ≤ 159.35		>159.35	
Non-HTN	1 [Reference]	—	1.316 (0.943, 1.836)	0.107	1.129 (0.802, 1.589)	0.487	1.182 (0.829, 1.684)	0.355
HTN	1 [Reference]	—	1.031 (0.819, 1.299)	0.794	1.033 (0.820, 1.300)	0.783	1.135 (0.889, 1.451)	0.310

HTN, hypertension; OR, odds ratio; CI, confidence interval; NLR, neutrophil-to-lymphocyte ratio; MLR, monocyte-to-lymphocyte ratio; NMLR, neutrophil-to-monocyte-to-lymphocyte ratio; SIRI, systemic inflammation response index; SII, systemic immune-inflammation index; AISI, aggregate index of systemic inflammation; PLR, platelet-to-lymphocyte ratio.

## Discussion

This study explored the correlation between different systemic inflammation indices and carotid plaques in patients with ACS. The results demonstrated that MLR, SIRI, and AISI were positively correlated with carotid plaques, while NLR, NMLR, SII, and PLR were not associated with carotid plaques.

Ischemic stroke caused by atherosclerotic internal carotid artery stenosis is currently one of the leading causes of death and disability worldwide (1). However, effective serum indices for the early identification of carotid plaques are still lacking (2). As novel serum bioparameters, non-coding RNAs are associated with carotid plaque formation and prognosis. It has been shown that maximum plaque thickness and intima-media thickness in patients with carotid atherosclerotic plaques are positively correlated with serum lncRNA CCAT2 levels, but negatively correlated with miRNA-216b levels (8). An additional study showed significant upregulation of miR-193b-5p, miR-193a-5p, and miR-125a-3p in carotid plaque patients compared with healthy controls, and these microRNAs were associated with inflammation and vascular growth, all of which are key factors in atherosclerosis and plaque formation (10). However, studies investigating the association between non-coding microRNAs and carotid plaque development and vulnerability have presented inconsistent findings (11). In addition, other indicators also demonstrated a correlation with carotid plaques. A Japanese study reported an association between carotid plaque and a TyG index of less than 9.06 (9, 21). Inflammation, as one of the important pathological processes involved in carotid plaque formation, has become a candidate target for the diagnosis and treatment of carotid atherosclerosis. Serum hs-CRP levels have been identified as a risk marker of carotid plaque, as shown by a 24% increased risk for each unit increase in hs-CRP (12, 22–24). In the present study, we explored the association of systemic inflammation indices with carotid plaque, providing a new direction for the discovery of carotid plaque in clinical practice.

Inflammation is involved in the formation and progression of a variety of diseases. Clinically, common blood routine tests—including white blood cells, lymphocytes, monocytes, and neutrophils—are associated with inflammation. In recent years, novel systemic inflammation indices derived from routine blood cell counts-including neutrophil-to-lymphocyte ratio (NLR), monocyte-to-lymphocyte ratio (MLR), neutrophil-to-monocyte-to-lymphocyte ratio (NMLR), systemic inflammatory response index (SIRI), systemic immune-inflammation index (SII), aggregate index of systemic inflammation (AISI), and platelet-to-lymphocyte ratio (PLR)-have demonstrated diagnostic and prognostic value in atherosclerosis-related diseases (14, 15). Studies have shown that NLR, MLR, NMLR, SIRI, and AISI are all positively associated with coronary artery calcification (CAC) in ACS (19). SII is also an effective tool for predicting morbidity and all-cause mortality in breast cancer patients (20). In chronic kidney disease (25, 26), MLR is positively associated with mortality risk and has a higher predictive validity for mortality risk than other clinical indicators (27). In the present study, we comprehensively analyzed the association of systemic inflammation indices—including NLR, MLR, NMLR, SIRI, SII, AISI, and PLR—with carotid plaques in patients with ACS. Our findings demonstrated that MLR, SIRI, and AISI were positively associated with the presence of carotid plaques, which is consistent with previous studies (13, 27). However, NLR, SII, and PLR have also been shown to be associated with carotid plaque in Ma's study (13), which is inconsistent with this study, possibly because our study population included ACS patients.

Compared with the findings reported by Ma et al. (13), discrepancies were observed in the associations of NLR, SII, and PLR with carotid plaques in the present study, which may be attributable to differences in study populations and study design. Ma et al. investigated a broader population of patients with coronary artery disease, whereas the current study focused specifically on patients with ACS, among whom the proportion of ST-segment elevation myocardial infarction (STEMI) was relatively high. It has been shown that STEMI is accompanied by pronounced ischemia–reperfusion injury, which triggers marked neutrophil mobilization and a heightened systemic inflammatory response. This process may alter the distribution and behavior of neutrophil- or platelet-based indices, such as NLR, SII, and PLR. In addition, differences in the timing of inflammatory index assessment may also contribute to the inconsistency. The analysis by Ma et al. was largely based on measurements obtained during relatively stable or non-acute phases, whereas in the present study, inflammatory indices were primarily derived from samples collected at admission during the acute phase of ACS. Given the dynamic nature of the acute inflammatory response, these indices may preferentially reflect short-term stress reactions rather than the chronic atherosclerotic burden.

In our ACS cohort, monocyte-containing inflammatory indices—including MLR, SIRI, and AISI—showed significant associations with carotid plaques, whereas traditional ratio-based indices such as NLR, SII, and PLR did not. This finding suggests that under the high inflammatory burden of ACS, monocyte-mediated inflammation may be more closely linked to the underlying pathophysiological processes than indices dominated by neutrophil or platelet components. As key effectors of the innate immune system, monocytes play a central role in the initiation and progression of atherosclerosis by infiltrating the vascular wall, differentiating into macrophages, promoting lipid uptake, releasing pro-inflammatory cytokines, and contributing to plaque growth and instability. In contrast, indices such as NLR, SII, and PLR primarily reflect the relative balance between neutrophils, platelets, and lymphocytes, which may be more indicative of acute stress-related leukocyte responses rather than chronic inflammation driving atherosclerotic plaque accumulation. Furthermore, during the chronic inflammatory phase following ACS, or in specific patient phenotypes, sustained recruitment and activation of monocytes and their macrophage derivatives represent a key pathological process in atherosclerosis, making monocyte-based composite indices such as MLR, SIRI, and AISI potentially more sensitive and stable markers of long-term inflammatory burden.

In the present study, inflammation indices that incorporate a monocyte component—such as MLR, SIRI, and AISI—were significantly associated with carotid plaque burden, whereas indices that do not include or only weakly reflect monocyte-related inflammation did not show consistent associations. These findings suggest that monocyte-driven inflammatory responses may play an important role in the progression of atherosclerosis and plaque instability following ACS (28, 29). Monocytes are key mediators of vascular inflammation and contribute to macrophage accumulation within the arterial wall, foam cell formation, and plaque destabilization. The stronger associations observed for monocyte-related inflammatory indices support the concept that innate immune activation, particularly monocyte–macrophage-mediated inflammation, plays a critical role in vascular remodeling after ACS.

Subgroup analyses showed that the significant associations between systemic inflammation indices and carotid plaques were mainly observed in women and in non-diabetic patients, suggesting both biological and clinical explanations. With respect to sex differences, estrogen has been reported to exert anti-inflammatory effects and to promote plaque stabilization, making women potentially more sensitive to subtle changes in inflammatory activity. As a result, even mild elevations in systemic inflammation may better reflect plaque-related inflammatory burden in female patients. In contrast, men generally exhibit a higher baseline inflammatory status and a greater prevalence of traditional risk factors such as smoking, alcohol consumption, and dyslipidemia, which may attenuate the discriminative value of a single inflammation-based index. In addition, carotid plaques in women tend to be more inflammation-driven, whereas plaques in men are more strongly associated with lipid accumulation and long-term exposure to conventional risk factors, rendering inflammation a relatively less dominant contributor.

In diabetes-stratified analysis, patients with diabetes typically exhibit a chronic low-grade inflammatory state, characterized by persistently elevated levels of inflammatory mediators such as CRP and TNF-α. This sustained background inflammation may lead to a “baseline saturation” effect, thereby limiting the ability of inflammation-derived indices to further discriminate plaque risk. Moreover, carotid plaque development in diabetic patients is more strongly driven by metabolic abnormalities, advanced glycation end-product accumulation, oxidative stress, and endothelial dysfunction, rather than by inflammation alone (30). By contrast, in non-diabetic patients, systemic inflammatory levels show a wider dynamic range, and elevations in inflammatory indices are more likely to directly reflect plaque-related pathological processes, resulting in a more pronounced association in this subgroup.

### Limitations

Several limitations of this study should be acknowledged. First, this was a retrospective single-center study, which may introduce selection bias and limit the generalizability of the findings to broader populations. Second, detailed characteristics of carotid plaques—such as echogenicity, plaque composition, and degree of luminal stenosis—were not available. These features are clinically more informative than the mere presence or absence of plaque and could provide additional insights into plaque vulnerability and risk stratification. Third, the exact timing of blood sampling for inflammatory indices relative to the onset of ACS, as well as information on concurrent medical treatments—particularly statins and anti-inflammatory therapies—was not available. These factors may substantially influence inflammatory marker levels and could potentially confound the observed associations. Finally, the systemic inflammation indices analyzed in this study were derived from peripheral blood cell counts rather than from direct measurements of immune cell subsets or circulating cytokines, which limits the ability to fully capture the underlying inflammatory biology.

## Conclusion

In patients with ACS, systemic inflammation parameters MLR, SIRI and AISI were positively correlated with carotid plaques, which may represent a new target for early diagnosis and treatment of carotid plaques in ACS. Subgroup analyses further indicated that these associations were predominantly observed in women and in non-diabetic patients.

## Data Availability

The raw data supporting the conclusions of this article will be made available by the authors, without undue reservation.
