# Supplementary material for: Association between systemic inflammation indices and carotid plaques in acute coronary syndrome
Source: Front Cardiovasc Med. 2026 Apr 1;13:1762926. doi: 10.3389/fcvm.2026.1762926 (PMC13079013; doi:10.3389/fcvm.2026.1762926)
Supplement: Supplementary file 1 [file table1.docx]

**Supplementary Table** Association between systemic inflammation parameters and baseline characteristics

|  | NLR |  | MLR |  | NMLR |  | SIRI |  | SII |  | AISI |  | PLR |  |
| --- | --- | --- | --- | --- | --- | --- | --- | --- | --- | --- | --- | --- | --- | --- |
|  | r | P | r | P | r | P | r | P | r | P | r | P | r | P |
| Gender, F/M | 0.204 | <0.001*** | 0.305 | <0.001*** | 0.218 | <0.001*** | 0.283 | <0.001*** | 0.091 | <0.001*** | 0.185 | <0.001*** | -0.032 | 0.007** |
| Age | 0.132 | <0.001*** | 0.171 | <0.001*** | 0.139 | <0.001*** | 0.115 | <0.001*** | 0.076 | <0.001*** | 0.073 | <0.001*** | 0.102 | <0.001*** |
| Hypertension | 0.038 | 0.001** | 0.004 | 0.745 | 0.035 | 0.003** | 0.044 | <0.001*** | 0.062 | <0.001*** | 0.061 | <0.001*** | 0.022 | 0.06 |
| Diabetes | -0.017 | 0.158 | -0.052 | <0.001*** | -0.021 | 0.077 | 0.007 | 0.539 | -0.001 | 0.926 | 0.016 | 0.184 | -0.063 | <0.001*** |
| Hyperlipidemia | -0.089 | <0.001*** | -0.093 | <0.001*** | -0.091 | <0.001*** | -0.075 | <0.001*** | -0.063 | <0.001*** | -0.058 | <0.001*** | -0.067 | <0.001*** |
| Smoking | 0.108 | <0.001*** | 0.168 | <0.001*** | 0.117 | <0.001*** | 0.189 | <0.001*** | 0.059 | <0.001*** | 0.142 | <0.001*** | -0.06 | <0.001*** |
| Drinking | 0.073 | <0.001*** | 0.142 | <0.001*** | 0.081 | <0.001*** | 0.136 | <0.001*** | 0.03 | 0.010* | 0.096 | <0.001*** | -0.04 | 0.001** |
| AST | 0.013 | 0.281 | 0.067 | <0.001*** | 0.019 | 0.111 | 0.044 | <0.001*** | -0.022 | 0.059 | 0.015 | 0.218 | -0.042 | <0.001*** |
| ALT | -0.02 | 0.093 | -0.005 | 0.696 | -0.019 | 0.106 | 0.029 | 0.014* | -0.029 | 0.015* | 0.017 | 0.146 | -0.1 | <0.001*** |
| CREA | 0.199 | <0.001*** | 0.253 | <0.001*** | 0.208 | <0.001*** | 0.257 | <0.001*** | 0.126 | <0.001*** | 0.192 | <0.001*** | 0.014 | 0.248 |
| UA | 0.026 | 0.031* | 0.064 | <0.001*** | 0.029 | 0.012* | 0.101 | <0.001*** | -0.006 | 0.628 | 0.068 | <0.001*** | -0.113 | <0.001*** |
| TC | -0.142 | <0.001*** | -0.198 | <0.001*** | -0.151 | <0.001*** | -0.151 | <0.001*** | -0.037 | 0.002** | -0.07 | <0.001*** | -0.005 | 0.699 |
| TG | -0.141 | <0.001*** | -0.184 | <0.001*** | -0.149 | <0.001*** | -0.092 | <0.001*** | -0.063 | <0.001*** | -0.04 | 0.001** | -0.134 | <0.001*** |
| LDL-C | -0.096 | <0.001*** | -0.14 | <0.001*** | -0.103 | <0.001*** | -0.099 | <0.001*** | -0.005 | 0.691 | -0.029 | 0.015* | 0.018 | 0.137 |
| HDL-C | -0.11 | <0.001*** | -0.153 | <0.001*** | -0.116 | <0.001*** | -0.16 | <0.001*** | -0.063 | <0.001*** | -0.116 | <0.001*** | 0.019 | 0.104 |
| LP(a) | 0.065 | <0.001*** | 0.045 | <0.001*** | 0.064 | <0.001*** | 0.056 | <0.001*** | 0.102 | <0.001*** | 0.083 | <0.001*** | 0.101 | <0.001*** |
| GLU | 0.086 | <0.001*** | -0.014 | 0.245 | 0.078 | <0.001*** | 0.075 | <0.001*** | 0.094 | <0.001*** | 0.083 | <0.001*** | <0.001 | 0.973 |
| HCY | 0.191 | <0.001*** | 0.227 | <0.001*** | 0.199 | <0.001*** | 0.23 | <0.001*** | 0.137 | <0.001*** | 0.181 | <0.001*** | 0.055 | <0.001*** |
| BNP | 0.177 | <0.001*** | 0.222 | <0.001*** | 0.185 | <0.001*** | 0.196 | <0.001*** | 0.111 | <0.001*** | 0.142 | <0.001*** | 0.068 | <0.001*** |
| hsTnI | 0.226 | <0.001*** | 0.247 | <0.001*** | 0.233 | <0.001*** | 0.263 | <0.001*** | 0.162 | <0.001*** | 0.209 | <0.001*** | 0.05 | <0.001*** |
| CK-MB | 0.126 | <0.001*** | 0.139 | <0.001*** | 0.13 | <0.001*** | 0.145 | <0.001*** | 0.077 | <0.001*** | 0.105 | <0.001*** | 0.018 | 0.128 |
| D-dimer | 0.186 | <0.001*** | 0.175 | <0.001*** | 0.189 | <0.001*** | 0.178 | <0.001*** | 0.163 | <0.001*** | 0.16 | <0.001*** | 0.125 | <0.001*** |

**Abbreviations**: WBC, white blood cell; RBC, red blood cell; LYM, lymphocyte; MONO, monocyte; NEUT, neutrophile; PLT, platelets; AST, Aspartate aminotransferase; ALT, Alanine Aminotransferase; CREA, Creatinine; UA, uric acid; CHOL, cholesterol; TG, triglyceride; LDL-C, low-density lipoprotein cholesterol; HDL-C, high-density lipoprotein cholesterol; LP (a), lipoprotein a; GLU, blood glucose; HCY, homocysteines; BNP, B-type natriuretic peptide; hsTnI, high-sensitivity Troponin I; CK-MB, Creatine Kinase-MB.
